# Leveraging Prior Healthy Participant Pharmacokinetic Data to Evaluate the Impact of Renal and Hepatic Impairment on Ritlecitinib Pharmacokinetics

**DOI:** 10.1208/s12248-023-00792-8

**Published:** 2023-03-28

**Authors:** Vivek Purohit, Yeamin Huh, Jessica Wojciechowski, Anna Plotka, Stephanie Salts, Jeremias Antinew, Angela Dimitrova, Timothy Nicholas

**Affiliations:** 1 Pfizer Inc, Groton, Connecticut USA; 2 Pfizer Inc, Collegeville, Pennsylvania USA; 3 Pfizer Inc, San Diego, California USA; 4 Pfizer Inc, Cambridge, Massachusetts USA

**Keywords:** hepatic, population pharmacokinetics, renal, ritlecitinib

## Abstract

**Abstract:**

Ritlecitinib is a selective, covalent, irreversible inhibitor of Janus kinase 3 (JAK3) and the tyrosine kinase expressed in hepatocellular carcinoma (TEC) family kinases. Pharmacokinetics and safety of ritlecitinib in participants with hepatic (Study 1) or renal (Study 2) impairment were to be characterized from two phase I studies. Due to a study pause caused by the COVID-19 pandemic, the study 2 healthy participant (HP) cohort was not recruited; however, the demography of the severe renal impairment cohort closely matched the study 1 HP cohort. We present results from each study and two innovative approaches to utilizing available HP data as reference data for study 2: a statistical approach using analysis of variance and an *in silico* simulation of an HP cohort created using a population pharmacokinetics (POPPK) model derived from several ritlecitinib studies. For study 1, the observed area under the curve for 24-h dosing interval and maximum plasma concentration for HPs and their observed geometric mean ratios (participants with moderate hepatic impairment *vs* HPs) were within 90% prediction intervals from the POPPK simulation-based approach, thereby validating the latter approach. When applied to study 2, both the statistical and POPPK simulation approaches demonstrated that patients with renal impairment would not require ritlecitinib dose modification. In both phase I studies, ritlecitinib was generally safe and well tolerated. These analyses represent a new methodology for generating reference HP cohorts in special population studies for drugs in development with well-characterized pharmacokinetics in HPs and adequate POPPK models.

**Trial Registration:**

ClinicalTrials.gov NCT04037865, NCT04016077, NCT02309827, NCT02684760, and NCT02969044.

**Graphical Abstract:**

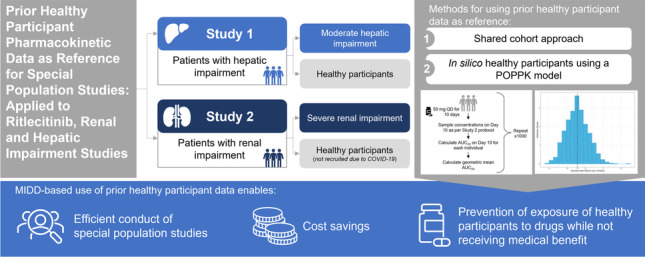

**Supplementary Information:**

The online version contains supplementary material available at 10.1208/s12248-023-00792-8.

## Introduction

Ritlecitinib is a selective, covalent, irreversible inhibitor of Janus kinase 3 and the tyrosine kinase expressed in hepatocellular carcinoma family kinases (1) currently in development for the treatment of alopecia areata (AA) (2), rheumatoid arthritis (RA) (3), vitiligo, ulcerative colitis, and Crohn’s disease (4). Ritlecitinib has completed a dose-ranging phase III trial for AA, and its registration applications for the dose of 50 mg are under review.

Three phase I studies (NCT02309827 (4), NCT02684760, and NCT03232905) in healthy participants (HPs) investigated oral ritlecitinib at single doses ranging from 5 to 800 mg and at multiple doses ranging from 50 to 400 mg once daily (QD) and 100 mg and 200 mg twice daily for up to 14 days. The pharmacokinetic (PK) profile of ritlecitinib was characterized by rapid absorption and elimination (terminal half-life, ≈2 h) with approximately dose-proportional exposures up to 200 mg with time-dependent changes in PK, especially at higher doses. Ritlecitinib is predominantly metabolized by multiple glutathione S-transferases and cytochrome P450 enzymes; <8% of ritlecitinib is excreted unchanged in urine after oral and intravenous administration. Ritlecitinib was well tolerated with an acceptable safety profile in HPs and participants with moderate to severe RA and AA (2, 3).

Here, we describe two phase I studies evaluating the PK, safety, and tolerability of multiple oral-dose administrations of ritlecitinib in participants with hepatic (study 1) and renal (study 2) impairment. The primary clearance mechanism for ritlecitinib is metabolism, and although the renal pathway may play a limited role in ritlecitinib excretion, renal impairment can impact drug PK through inhibition of hepatic and gut drug metabolism and transport pathways (5).

Phase I studies in special populations require a reference HP cohort with matched demographic characteristics. In study 1, HPs were enrolled for comparison with participants with hepatic impairment. Due to the COVID-19 pandemic, the matching HP cohort in study 2 was not recruited. The current analysis considers traditional and model-informed drug development (MIDD) approaches to use available HP data as reference data. The feasibility of the MIDD approach as a new methodology for generating reference HP cohorts in special population studies, when adequate HP data are already available in other studies, is examined.

## Methods

### Study Design

#### Study 1: Participants with Hepatic Impairment

Study 1 was a phase I, nonrandomized, open-label, multiple-dose, parallel-cohort, two-part study to investigate the effects of hepatic impairment on the plasma PK, safety, and tolerability of ritlecitinib. Part 1 planned to enroll ≈16 participants: eight with moderate hepatic impairment and eight HPs with normal hepatic function. Participants were aged 18‒70 years, body mass index (BMI) of  ≥17.5 to ≤40 kg/m^2^, and body weight of  >50 kg. Dependent on outcomes in part 1, part 2 planned to enroll ≈8 participants with mild hepatic impairment and would be conducted if the point estimate of ritlecitinib area under the plasma concentration–time curve (AUC) for 24-h dosing interval (AUC_0-24_) geometric mean ratio (GMR) for the moderate hepatic impairment cohort *versus* the HP cohort was  ≥2.0. Part 2 was not conducted as the criterion to progress was not met (the point estimate of the GMR was 1.19; see the “Results”).

Participants in the hepatic impairment cohort met the criteria for class B of the Child–Pugh classification (6) (moderate: 7–9 points), within 28 days of ritlecitinib administration. They had stable hepatic impairment, defined as no clinically significant change in disease status within 30 days prior to screening, and had a diagnosis of hepatic dysfunction due to hepatocellular disease.

HPs had no known or suspected hepatic disease and no evidence or history of clinically significant hematologic, renal, endocrine, pulmonary, gastrointestinal, cardiovascular, hepatic, psychiatric, neurologic, dermatologic, or allergic disease. HPs were enrolled following participants with moderate hepatic impairment and matched for mean age (± 10 years), weight (± 15 kg), race, and sex (± 2 participants per sex) of the moderate impairment cohort.

Participants received oral doses of ritlecitinib 30 mg QD under nonfasting conditions for 9 days. On day 10, participants received ritlecitinib 30 mg following an 8-h fast. Blood samples were collected predose on days 7, 8, 9, and 10 for assessment of ritlecitinib minimum plasma concentration, and at 0.5, 1, 2, 3, 4, 6, 8, 12, 14, and 24 h postdose on day 10 for PK assessments (AUC_0-24_ and maximum observed plasma concentration [*C*_max_]). Multiple doses were used to allow ritlecitinib concentrations to achieve steady state. PK parameters were calculated for each participant and each treatment using noncompartmental analysis (NCA) of concentration–time data. Samples below the lower limit of quantification were set to zero. The study design was consistent with guidance for PK studies in patients with impaired hepatic function (7).

All-causality and treatment-related treatment-emergent adverse events (TEAEs) were monitored throughout the study and via telephone follow-up 28 + 3 days after last dose administration. Clinical laboratory tests, vital sign assessments, and physical examinations were performed during screening and days specified in the protocol.

#### Study 2: Participants with Renal Impairment

Study 2 was a phase I, nonrandomized, open-label, multiple-dose, parallel-cohort, multisite, two-part study to investigate the effect of renal impairment on the plasma PK, safety, and tolerability of ritlecitinib. Part 1 planned to enroll ≈8 participants with severe renal impairment and 8 HPs with normal renal function. Approximately 8 participants with moderate renal impairment and 8 with mild renal impairment were to be enrolled in part 2. Participants in the severe renal impairment group were recruited first: HPs were to be recruited later so that mean age (± 10 years) and weight (± 15 kg) could be matched. Following part 1, part 2 would be conducted if the point estimate of ritlecitinib AUC_0-24_ GMR for the severe renal impairment cohort *versus* the HP cohort was  >2.0.

Participants were aged 18‒75 years, with BMI of  ≥17.5 to ≤40 kg/m^2^ and body weight of  >50 kg. Participants in the severe renal impairment cohort met criteria for severe renal impairment based on the Modification of Diet in Renal Disease equation (5) (estimated glomerular filtration rate [eGFR]  <30 mL/min but not requiring hemodialysis) but were otherwise in good general health commensurate with the population with renal impairment. Participants in the HP cohort had no clinically relevant abnormalities and eGFR  ≥90 mL/min.

Participants received oral doses of ritlecitinib 50 mg QD under nonfasting conditions for 9 days. On day 10, participants received ritlecitinib 50 mg following a fast of  ≥10 h. Blood samples were collected to assess ritlecitinib concentration predose on days 8, 9, and 10, and 0.25, 0.5, 1, 2, 4, 6, 8, 12, 16, and 24 h postdose on day 10. Steady-state (day 10) AUC_0-24_ and *C*_max_ were calculated using the NCA approach. The study design was consistent with guidance for PK studies in patients with impaired renal function (8).

TEAEs were monitored throughout the study and by telephone follow-up 28‒35 days after last dose. Physical examinations and vital signs assessments were performed during screening and on days specified in the protocol. Clinical laboratory tests and eGFR measurements were conducted during two screening visits, on day 1, and on days 2, 5, 8, and 11 (at the second screening visit, and on days 2 and 8, only serum creatinine for eGFR measurement was assessed).

### Estimation of Effect of Renal Impairment on Ritlecitinib Exposure

The severe renal impairment cohort in study 2 was completed; however, the study was paused due to the COVID-19 pandemic, and the HP cohort for part 1 was not enrolled. HP data were obtained from available data of completed phase I studies using two different approaches: (1) HP data from completed study 1 (shared cohort approach) and (2) *in silico* HP cohorts derived from a population PK (POPPK) model.

#### Analysis of Variance Approach

A statistical approach using analysis of variance (ANOVA; SAS v9.4) was applied to compare the natural log-transformed ritlecitinib AUC_0-24_ and *C*_max_ of the various cohorts. For study 1, the HP (reference) and the moderate hepatic impaired function (test) cohorts were compared. For study 2, the HP cohort (shared cohort) from study 1 (reference) and the severe impaired renal function cohort from study 2 (test) were compared. The HP cohort from study 1 was selected as a reference group because the cohort demographics closely matched that of the severe renal impairment cohort from study 2. HPs with eGFR  <90 mL/min were excluded from the analysis to meet the study 2 inclusion/exclusion criteria. This assessment was consistent with traditional methods as described in US Food and Drug Administration guidance for studies in patients with impaired renal function (8). Since systemic exposures of ritlecitinib increase linearly from 30- to 50 -mg doses, the PK parameters (AUC_0-24_ and *C*_max_) for HP cohort from study 1 were normalized to the 50-mg dose administered in study 2 for analyses.

Estimates of adjusted mean differences (test − reference) and corresponding 90% confidence intervals (CIs) were obtained from the model. The mean differences and 90% CIs for the differences were exponentiated to provide estimates of the ratio of geometric means (test/reference) and 90% CIs for the ratios.

#### Clinical Trial Simulation Approach

##### Population Pharmacokinetic Model

**Fig. 1:**
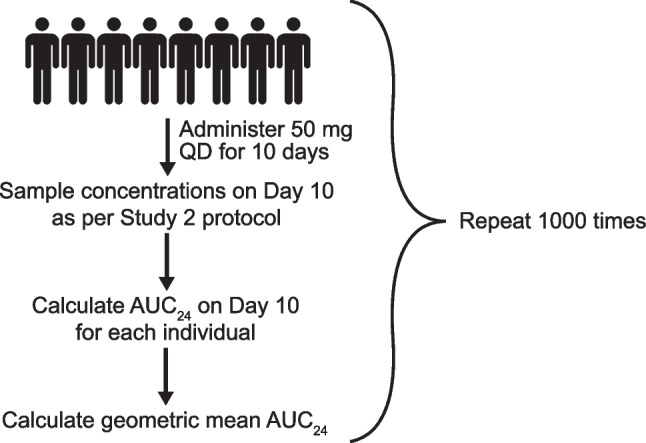
Simulation and analysis of an *in silico* cohort. AUC_24_, area under the plasma concentration–time curve for 24-h dosing interval; QD, once daily

To simulate *in silico* HP cohorts, a POPPK model for ritlecitinib was developed using pooled data from two phase I studies in HPs (NCT02309827 and NCT02684760) and one phase II study in patients with RA (NCT02969044) (Fig. 1). A POPPK model had been previously developed using NONMEM® and standard nonlinear mixed-effect modeling procedures and goodness-of-fit diagnostics (Text S1 and Figs. 1–3 of Supplementary Material).

The final model was a two-compartment model with first-order oral absorption, interindividual variance on apparent clearance (CL/F) and apparent central volume of distribution (Vc/F), and a proportional residual error model. Covariates incorporated into the model included allometric scaling on CL/F and Vc/F referenced to a 75-kg individual with exponents of 0.75 and 1, respectively; effect of patients with RA on CL/F; effect of food on first-order absorption rate constant; and effect of total daily doses  >100 mg on CL/F. There was no apparent difference in clearance with respect to age.

The simulation was conducted in two parts independently for study 1 and study 2. The first part simulated PK parameters of demographically matched HP cohorts for the completed study 1, which served as an external validation of the simulation approach. The second part applied the simulation to study 2. The 1000 trials were simulated and for each study, the *in silico* HP cohort consisting of eight individuals with PK parameters was created by randomly generated weights from a uniform distribution between 69.9 and 99.9 kg (study 1) or 73 and 103 kg (study 2). Individual random-effect parameters for CL/F and Vc/F were drawn from the variance–covariance matrix described by the POPPK model. Each simulated individual was administered their respective study dose for 10 days to reflect actual study conduct. Simulated concentrations (individual predictions with residual unexplained variance applied) were sampled on day 10 following the study schedule. Those below the lower limit of quantification were set to zero. AUC_0-24_ was calculated for each individual based on the linear-up/log-down NCA approach using the PKNCA package (v0.9.1) in R (v3.6.1).

##### Comparison with Hepatic and Renal Impairment Cohort

To compare simulated HP reference data with the study 1 (moderate hepatic impairment) and study 2 (severe renal impairment) cohorts, the GMRs (90% CI) for AUC_0-24_ and *C*_max_ were calculated for each *in silico* HP cohort relative to their respective impairment cohort using ANOVA. Results for the 1000 trials were summarized by median GMR (and nonparametric 90% prediction interval [PI] of all GMRs), mean GMR (and mean lower and upper 90% CI derived from ANOVA results), and proportion of trials with a GMR of  ≥2.0.

### Ethics

All study protocols were reviewed and approved by each site’s institutional review board or ethics committee and conducted in accordance with the Declaration of Helsinki and in compliance with all International Council for Harmonisation Good Clinical Practice Guidelines. All participants provided written, informed consent.

## Results

### Study 1

#### Participants

Ten participants with moderate hepatic impairment and eight HPs were enrolled and received ritlecitinib (Table I). Demographic characteristics for HPs were comparable to those for participants with moderate hepatic impairment. Most participants were White (*n*=17/18), male (*n*=11/18), and aged 45‒64 years at baseline (*n*= 14/18). The mean (range) weight was 83.2 (66.9–107.9) kg; mean (range) BMI was 29.9 (22.8–36.4) kg/m^2^.

**Table I:** Summary of Demographic and Baseline Characteristics: All Treated Participants

	Study 1		Study 2
Summary statistic	Moderate hepatic impairment (*n*=10)	Healthy participants (*n*=8)	Severe renal impairment (*n*=8)
Age, years			
18‒44, *n* (%)	0	0	1 (12.5)
45‒64, *n* (%)	7 (70.0)	7 (87.5)	4 (50.0)
≥65, *n* (%)	3 (30.0)	1 (12.5)	3 (37.5)
Mean (SD)	59.0 (6.9)	57.4 (6.5)	59.5 (9.8)
Male sex, *n* (%)	7 (70.0)	4 (50.0)	5 (62.5)
Race, *n* (%)			
White	10 (100)	7 (87.5)	7 (87.5)
Black or African American	0	1 (12.5)	1 (12.5)
Body mass index, mean (SD), kg/m^2^	31.1 (4.0)	28.4 (3.4)	30.5 (1.6)
Weight, mean (SD), kg	86.4 (14.8)	79.1 (8.7)	87.8 (4.9)

*SD* standard deviation

#### Pharmacokinetics

Sixteen participants were included in the PK parameter analysis: eight with moderate hepatic impairment and eight HPs (Table II). Following administration of multiple oral doses of ritlecitinib 30 mg QD, *C*_max_ was achieved with the median time to maximum concentration (*T*_max_) of 1 h in participants with moderate hepatic impairment *versus* 0.5 h in HPs. The respective AUC_0-24_ was 454.5 *versus* 383.6 ng·h/mL and *C*_max_ 194.3 *versus* 186.9 ng/mL in participants with moderate hepatic impairment *versus* HPs. The geometric mean CL/F of ritlecitinib was numerically lower in participants with moderate hepatic impairment (66.0 L/h) compared with HPs (78.2 L/h).

**Table II:** Descriptive Summary of Plasma Ritlecitinib Pharmacokinetic Parameters

	Study 1 (30 mg QD)		Study 2 (50 mg QD)
Parameter^a^	Moderate hepatic impairment (*n*=8)^b^	Healthy participants (*n*=8)	Severe renal impairment (*n*=8)
AUC_0-24_, ng·h/mL	454.5 (45)	383.6 (16)	986.3 (33)
AUC_last_, ng·h/mL	448.6 (46)	377.2 (17)	983.2 (33)
CL/F, L/h	65.96 (45)	78.20 (16)	50.63 (34)
*C*_max_, ng/mL	194.3 (49)	186.9 (24)	445.6 (21)
*T*_max_, h	1.00 (0.500‒2.00)	0.500 (0.500‒1.00)	0.50 (0.25‒1.00)

^*a*^Geometric mean (geometric % coefficient of variation) for all parameters except median (range) for *T*_max_

^*b*^Two enrolled participants were nonevaluable for PK

*AUC*_*0-24*_ area under the plasma concentration–time curve for 24-h dosing interval, *AUC*_*last*_ area under the plasma concentration–time curve from time 0 to the time of last measurable concentration, *C*_*max*_ maximum observed plasma concentration, *CL/F* apparent oral clearance, *PK* pharmacokinetics, *QD* once daily, *T*_*max*_ time to maximum plasma concentration

#### Safety

Treatment-related TEAEs were reported in four (40.0%) participants with moderate hepatic impairment and one (12.5%) HP (Table I of Supplementary Material). A serious AE, a severe AE, and TEAEs leading to discontinuation of study drug were reported in participants with moderate hepatic impairment (*n*=1 [10.0%], *n*=1 [10.0%], and *n*=2 [20.0%], respectively). Treatment discontinuations were due to thrombocytopenia and hepatic enzyme increased (*n*=1 each). No dose reductions or temporary discontinuations due to AEs or deaths were reported.

#### Statistical Comparison: Hepatic Impairment *versus* HP Cohorts

The ratio (90% CI) of the adjusted geometric mean for AUC_0-24_ for participants with moderate hepatic impairment (test) *versus* HPs (reference) was 118.50% (87.96–159.64%) (Table III). The ratio (90% CI) of the adjusted geometric mean for *C*_max_ for the test *versus* reference was 104.00% (74.48–145.23%), indicating that *C*_max_ values were comparable. Observed variability (geometric coefficient of variation [CV]%) values for ritlecitinib AUC_0-24_ (45% *vs* 16%) and C_max_ (49% *vs* 24%) were higher in participants with moderate hepatic impairment *versus* HPs.

**Table III:** Statistical Summary: One-Way ANOVA of Natural Log-Transformed Plasma Ritlecitinib Pharmacokinetic Parameters

Parameter	Geometric means				Adjusted geometric means^a^			
	Test: moderate hepatic impairment (study 1)	Reference: healthy participants (study 1)	Ratio (%) of geometric means	90% CI (%) for ratio	Test: severe renal impairment (study 2)	Reference: healthy participants (study 1)	Ratio (%) of adjusted means	90% CI (%) for ratio
AUC_0-24_, ng·h/mL	454.5	383.6	118.50	87.96–159.64	986.3	635.7	155.15	122.83–195.98
*C*_max_, ng/mL	194.3	186.9	104.00	74.48–145.23	445.6	308.4	144.48	114.24–182.73

^*a*^For comparison with study 2, values from study 1 have been adjusted to 50 mg QD for each participant

*ANOVA* analysis of variance, *AUC*_*0-24*_ area under the plasma concentration–time curve for 24-h dosing interval, *CI* confidence interval, *C*_*max*_ maximum observed plasma concentration, *QD* once daily

#### Clinical Trial Simulation Analysis: Application to Completed Study 1 in Participants with Hepatic Impairment

A summary of AUC_0-24_ and C_max_ for the *in silico* HP generated by the POPPK model and the observed moderate hepatic impairment cohort in study 1 are provided in Table IV. PK analyses demonstrated that the observed mean AUC_0-24_, *C*_max_, and GMRs in study 1 (Table III) were within the 90% PI of all simulated PK parameters and GMRs (Table IV, Fig. 4 of Supplementary Material). Of the 1000 *in silico* trials conducted, none exhibited a GMR of  ≥2.0 for AUC. Since the POPPK simulation analysis applied to the completed hepatic impairment study was consistent with the observed results in study 1, the result serves as an external validation of this model-informed approach.

**Table IV:** POPPK Simulation Analysis

Summary	AUC_0-24_ (ng·h/mL)	C_max_ (ng/mL)
Moderate hepatic impairment *versus* healthy participants		
*in silico* healthy participant cohorts (*n* = 1000)		
Median (90% PI) of geometric means	345 (309–388)	184 (152–222)
Moderate hepatic impairment cohort (*n* = 1)		
Geometric mean	455	194
Geometric mean ratio^a^		
Median (90% PI)^b^	1.32 (1.17–1.47)	1.06 (0.87–1.28)
Mean (90% CI)^c^	1.32 (0.98–1.78)	1.07 (0.75–1.53)
Trials with geometric mean ratio ≥2.0, % (*n* = 1000)	0	0
Severe renal impairment *versus* healthy participants		
*in silico* healthy participant cohorts (*n* = 1000)		
Median (90% PI) of geometric means	578 (515–650)	314 (262–372)
Severe renal impairment cohort (*n* = 1)		
Geometric mean	986	446
Geometric mean ratio		
Median (90% PI)^b^	1.71 (1.52–1.92)	1.42 (1.20–1.70)
Mean (90% CI)^c^	1.71 (1.35–2.17)	1.43 (1.14–1.80)
Trials with geometric mean ratio ≥2.0, % (*n* = 1000)	1	0.2

^*a*^Observed AUC_0-24_ geometric mean ratio was 1.19 and within the 90% PI

^*b*^Determined based on the non-parametric distribution of 1000 geometric mean ratios (test:reference)

^*c*^Determined based on the results of ANOVA in which the mean summary statistics of 1000 trials are presented (mean geometric mean ratio [mean lower 90% CI, mean upper 90% CI])

*ANOVA* analysis of variance, *AUC*_*0-24*_ area under the plasma concentration–time curve for 24-h dosing interval, *CI* confidence interval, *C*_*max*_ maximum plasma concentration, *PI* prediction interval, *POPPK* population pharmacokinetics

### Study 2

#### Participants

Eight participants with severe renal impairment were enrolled and received ritlecitinib (Table I). Most participants were White (*n*=7/8); median (range) age was 58.5 (43‒72) years. The mean weight (standard deviation [SD]) was 87.8 (4.9) kg and mean (SD) BMI was 30.5 (1.7) kg/m^2^.

#### Pharmacokinetics

After administration of multiple oral doses of ritlecitinib 50 mg QD, median *T*_max_ was 0.5 h. Systemic exposures as measured by geometric mean values for AUC_0-24_ and *C*_max_, respectively, were 986.3 ng·h/mL and 445.6 ng/mL (Table IV), and observed variability (geometric CV%) was 33% and 21%.

#### Safety

Two participants reported three treatment-related TEAEs (aspartate aminotransferase increased, alanine aminotransferase increased, and dizziness) (Table 2 of Supplementary Material). There were no serious or severe AEs or dose reductions or temporary or permanent discontinuations from treatment or the study due to AEs.

### Statistical Comparison: Severe Renal Impairment (Study 2) *versus* HP Cohort (Study 1)

Baseline demographics of the severe renal impairment cohort (study 2) and HPs from study 1 were adequately matched for age, weight, BMI, and female:male ratio (Table V). Two HPs from study 1 with eGFR  <90 mL/min were excluded from the PK parameter analysis. The ratio (90% CI) of the adjusted geometric mean for AUC_0-24_ for participants with severe renal impairment (study 2: test) *versus* HPs (study 1: reference) was 155.15% (122.83–195.98%) (Table III). For the test *versus* r﻿﻿﻿﻿eference cohorts, the ratio (90% CI) of the adjusted geometric mean for C_max_ was 144.48% (114.24–182.73%) (Table III). Using this approach, the GMR for AUC_0-24_ did not exceed 2.0; therefore, the criterion for conducting part 2 of study 2 to assess PK in participants with mild and moderate renal impairment was not met.

**Table V:** Comparison of Participant Demographics in Studies 1 and 2

Study	Participant cohort	n	Age, years^a^	Weight, kg^a^	BMI, kg/m^2a^	Female:male ratio, *n*
Study 2	Severe renal impairment	8	59.5 (9.8)	87.8 (4.9)	30.5 (1.6)	3: 5
Study 1	Healthy participant	6^b^	56.7 (7.3)	81.0 (9.4)	28.8 (3.5)	2: 4

^*a*^Mean (standard deviation)

^*b*^Two healthy participants from Study 1 with eGFR  <90 mL/min were excluded

*BMI* body mass index, *eGFR* estimated glomerular filtration rate

### Clinical Trial Simulation Analysis: Application to Study 2 in Renally Impaired Participants

A summary of AUC_0-24_ and *C*_max_ for the *in silico* HP and observed severe renal impairment cohorts is presented in Table IV. The median GMR of 1000 trials for AUC_0-24_ and C_max_, respectively, was 1.71 and 1.42, and 1.0% and 0.2% of the 1000 trials resulted in a GMR of  ≥2.0. The distribution of the GMRs is provided in Fig. 5 of Supplementary Material. Since the 90% PI for the median GMR did not exceed 2 for AUC_0-24_, it could be inferred that the GMR of severe renal impairment *versus* normal renal function was  <2.0. Hence, using the POPPK simulation analysis, the criterion for conducting part 2 of the study was also not met.

## Discussion

This study evaluated the PK, safety, and tolerability of ritlecitinib in phase I studies in participants with hepatic and renal impairment. In both studies, ritlecitinib was generally safe and well tolerated.

In the absence of an HP cohort to estimate the effect of renal impairment on ritlecitinib exposure, two approaches were taken. First, a statistical approach used the shared HP cohort from study 1 as a reference. The demographic characteristics of HPs from study 1 closely matched the renal impairment cohort in study 2. Hence, recruitment of a separate HP cohort in study 2 was not necessary as the available data satisfied the matching criteria for the HP cohort. Based on the estimated AUC_0-24_ GMR of 155.15% (90% CI, 122.83–195.98%), part 2 of the study was not conducted. Based on a log-normal distribution and observed variability of ritlecitinib, AUC_0-24_ within 0.5- to 2-fold of the mean is considered a typical range of exposures. Hence, an average 55% higher exposure expected in participants with renal impairment is not considered clinically significant and, with this approach, no dose adjustment would be necessary.

In contrast to the statistical approach, which relies on a single subset of participants with matched demographics, the POPPK simulation approach used a POPPK model developed using available data at the time of the analysis. The POPPK model characterized the PK of ritlecitinib with weight as the only significant covariate. This approach leveraged the ability of random sampling, uncertainty of exposures in HPs, and simulation of multiple matched HP cohorts to generate a distribution of possible outcomes. Hence, it provides a more robust estimation of the difference between populations than a traditional matched cohort study, as it uses data from a larger number of HPs than typically recruited for organ impairment studies. Simulations based on this POPPK model adequately represented the distribution of exposures that could be expected in a HP cohort. Relative to a traditional study design, in which a single HP cohort reference represents a random sample of 1, the POPPK simulation approach can generate thousands of reference groups, providing a more complete assessment of the likely distribution of the GMR.

The POPPK simulation approach was validated using the results from study 1. The estimated GMR (AUC_0-24_) for comparison between moderate hepatic impairment *versus* normal hepatic function (HP) groups in study 1 was 1.32 (90% PI, 1.17–1.47), for which the observed GMR of 1.19 was contained within the 90% PI. The POPPK simulation approach also led to the same decision not to proceed to part 2 of the study as in the completed study 1 and to the conclusion that no dose adjustment due to hepatic impairment would be necessary. This exercise validated the POPPK simulation approach used in study 2.

When applied to study 2, the POPPK simulation approach estimated the AUC_0-24_ in participants with severe renal impairment to be 71% higher relative to HPs. The less than two-fold higher systemic exposure is not considered clinically significant because it is within the typical range of exposures (0.5- to 2-fold) expected for a given dose of ritlecitinib. Hence, the POPPK simulation approach demonstrated that dose modification would not be required for participants with renal impairment.

Although there are a few examples that used historical HP data (9, 10), to the best of our knowledge, the *in silico* cohort is the first application of using data simulated from a POPPK model to analyze and interpret dedicated special population studies. A survey of recently approved New Drug Applications (2020–2021) revealed examples of POPPK-based post hoc assessment of special population studies in which matched HPs were recruited and POPPK-based assessment of patients with organ impairment included in phase II or phase III studies. POPPK-based assessment of the impact of organ impairment in patients included in phase II or phase III studies is consistent with US Food and Drug Administration guidance (8). However, it is limited to assessment of populations with mild organ impairment; those with moderate or severe organ impairment are rarely included in double-blind, placebo-controlled studies due to uncertainty of the correct dose and risk of higher than the targeted exposures, hence requiring dedicated studies with matched HP cohorts. The survey of recently approved New Drug Applications did not reveal any applications of POPPK-generated HP data to analyze and interpret dedicated special population studies.

There are limitations to our study. The methodology was applied to a drug that does not have a single route of metabolism and has a low fraction excreted unchanged in urine. This approach has not been applied to a drug with a single primary route of metabolism/excretion, which could determine whether this methodology can appropriately identify if a dose change for a special population is recommended due to increased exposure.

Ritlecitinib is predominately eliminated by metabolism, and the variability in PK could be explained by body weight only. However, for a drug primarily cleared by renal elimination, PK variability may be associated with other demographic factors, such as age and eGFR. The HP cohort for hepatic/renal impairment studies often includes elderly participants for demographic matching to minimize impact of intrinsic factors on PK. Since HP PK is usually characterized in younger participants in early-stage drug development, available PK data for the POPPK model to characterize PK across a wide age range may be limited and impact capacity to simulate a reliable HP cohort. Conversely, due to higher clearance relative to older participants, HP PK in younger participants provides a more conservative assessment of the likelihood for need of dose adjustments in special populations. This is specifically relevant when the conclusion is that dose adjustments are not necessary in special populations based on comparison with a younger HP cohort.

Since a formal sensitivity and specificity assessment of the methodology was not performed, application of this approach to historical data and other drugs will provide an overall insight of its utility.

One thousand simulations of *in silico* cohorts based on the POPPK model accounted for the variability observed when small sample sizes were enrolled and permitted determination of an empirical probability of trials that would result in recommended dose changes. The POPPK model presented should be considered an iteration of the model-development life cycle and would be expected to evolve according to that and its utility. Confirmation using the final POPPK model and formal covariate analysis with additional accrued patient data should be considered and planned before finalizing dosing recommendations in special populations (8). A final POPPK model (to be published) for ritlecitinib has confirmed the above results where the estimates for the moderate hepatic impairment and severe renal impairment *versus* HPs were 1.30 and 1.41, respectively.

## Conclusions

The MIDD approach described here is a novel method for using *in silico* and historical HP data to estimate relative systemic exposures in participants with renal impairment. The analyses represent a new standard for generating reference HP cohorts in special population studies for drugs in development with adequately characterized PK in HPs and adequately qualified POPPK models. The POPPK approach required a model to be available by this stage of clinical drug development, thus demonstrating the utility of having such models available early in drug development. The approaches presented here may be applied to any special population study, even in the absence of a disruptive event such as the COVID-19 pandemic. Although there is limited regulatory precedent for such approaches, advantages of this approach to utilizing available PK data include efficient conduct of special population studies, cost savings, and prevention of exposure of HPs to drugs while not receiving medical benefit.

## Supplementary Information

Below is the link to the electronic supplementary material.

Supplementary file1 (DOCX 795 KB)

## Data Availability

Upon request and subject to review, Pfizer will provide the data that support the findings of this study. Subject to certain criteria, conditions, and exceptions, Pfizer may also provide access to the related individual de-identified participant data. See https://www.pfizer.com/science/clinical-trials/trial-data-and-results for more information.
