# Supplementary material for: Leveraging Prior Healthy Participant Pharmacokinetic Data to Evaluate the Impact of Renal and Hepatic Impairment on Ritlecitinib Pharmacokinetics
Source: AAPS J. 2023 Mar 28;25(3):32. doi: 10.1208/s12248-023-00792-8 (PMC10047454; doi:10.1208/s12248-023-00792-8)
Supplement: Supplementary file 1 — Supplementary file1 (DOCX 795 KB) [file 12248_2023_792_MOESM1_ESM.docx]

# SUPPLEMENTARY MATERIALS

# Supplementary Text S1. Population PK Model METHODS

**Analysis Population**

Population pharmacokinetic (POPPK) model development for ritlecitinib used pooled data from two phase I studies in healthy participants (HPs) (NCT02309827 and NCT02684760) and one phase II study in patients with rheumatoid arthritis (RA) (NCT02969044). A summary of doses and ritlecitinib concentration sampling are provided in Table A.

**Table A. Summary of Studies Included in the POPPK Modeling Analysis**

| **Study Phase (NCT identifier)** | **Protocol Design** | **Population (n)** | **Dose Administration** | **Plasma Sampling** |
| --- | --- | --- | --- | --- |
| Phase I (NCT02309827) | Randomized, double-blind, third-party open, placebo- controlled, single, and multiple dose escalation, parallel-group study to investigate safety, tolerability, PK, and PD of ritlecitinib in HPs | HP (80) | SAD: Single doses of 5, 20, 50, 100, 200, 400,  and 800 mg ritlecitinib or placebo  MAD: Repeated doses of 50, 200, or 400 mg  QD, or 100 or 200 mg  BID for 14 days (fasted) | SAD: 0 (predose), 0.5,  1, 2, 4, 6, 8, 12, 16, 24,  36, and 48 h postdose MAD: Predose on Days 1, 4, 6, 8, 10, 12, and  14; 0.5, 1, 2, 4, 6, 8, 12,  16, 24, 36, and 48 h  postdose on Day 14 |
| Phase I (NCT02684760) | Open-label, single dose, 3-way crossover study to evaluate relative bioavailability of solid dose formulation of ritlecitinib under fasting conditions, and effect of a high-fat meal on bioavailability of solid dose  formulation of ritlecitinib in HPs | HP (14) | 50 mg ritlecitinib tablet under fasted conditions; 50 mg ritlecitinib oral solution under fasted conditions; and 50 mg ritlecitinib tablet under fed conditions, each as  a single dose | 0 (predose), 0.25, 0.5,  1, 2, 4, 6, 8, 12, 16, 24,  36, and 48 h postdose |
| Phase II (NCT02969044) | Randomized, double-blind, parallel-group, placebo- controlled, multicenter study to assess the efficacy and safety profile of ritlecitinib in participants with moderate to severe active RA with an inadequate response to  methotrexate | RA (70) | 200 mg ritlecitinib QD or matching placebo for 8 weeks (fasted) | 0 h (predose) on Days 1, 8, 15, 29, 43, and 57;  0.5 h postdose on Days 15, 29, 43, and 57; 1 h  postdose on Days 15, 43, and 57; 2 and 4 h postdose on Day 57 |

BID, twice daily; HP, healthy participant; MAD, multiple ascending dose; PD, pharmacodynamics; PK, pharmacokinetics; POPPK, population pharmacokinetics; QD, once daily; RA, rheumatoid arthritis; SAD, single ascending dose

# Model Development

Model development used NONMEM® Version VII Level 3.0. Population parameter estimation used first-order conditional estimation method with interaction (FOCEI) and individual parameters were obtained from empirical Bayes estimates. The ADVAN4 subroutine for two- compartment models with first-order absorption. Ritlecitinib concentrations were log-transformed both sides. Statistical and graphical output were generated using the R programming and statistical language (R 3.2.2).

# Structural Model Development

Based on graphical exploratory assessment, two-compartment model structures with first-order absorption were evaluated. The structural model included the effect of body weight on pharmacokinetic (PK) parameters *a priori*. Allometric scaling on CL/F (apparent clearance) and Q/F (apparent inter-compartmental clearance), and Vc/F (apparent central volume) and Vp/F (apparent peripheral volume) was referenced to a 75-kg individual with exponents fixed to 0.75 and 1, respectively. Apparent non-linear CL/F was observed from noncompartmental analyses and was implemented by allowing CL/F to change for total daily doses greater than 100 mg.

# Random Effects Model Development

Inter-individual variability (IIV) was assumed to be log-normally distributed for PK parameters. A proportional residual error model was used to described random unexplained variability (RUV) in ritlecitinib concentrations.

# Covariate Model Development

The effect of food on the first-order absorption rate constant (ka) was assessed in participants who had high-fat meals recorded. Participants where food status was not captured were grouped with the fasted participants. Patient population (i.e., HPs vs. patients with RA) was

assessed on CL/F. The effect of formulation, study, and age were graphically evaluated by individual random effect parameters versus covariate plots.

The full POPPK model was developed using stepwise forward selection. A change in objective function value (OFV) by more than 3.84 (*P*=0.05) was used as selection criteria for covariate inclusion. The final POPPK model was to be determined from the full model by backward elimination based on OFV and goodness-of-fit diagnostic plots. A change in OFV by more than 6.63 (*P*=0.01) was used as criteria for covariate removal. The suitability of the final population PK model was confirmed using goodness-of-fit diagnostic plots.

# RESULTS

**Analysis Population**

A total of 1519 ritlecitinib PK concentrations from 113 subjects were used to generate the final POPPK model.

# Final Model

The final model was a two-compartment model with first-order oral absorption, IIV on CL/F and Vc/F, and a proportional residual error model. Covariates incorporated into the model included: allometric scaling on CL/F and Vc/F referenced to a 75-kg individual with exponents of 0.75 and 1, respectively; effect of patients with RA on CL/F; effect of food on first-order absorption rate constant; and effect of total daily doses >100 mg on CL/F. There was no apparent difference in clearance with respect to age. Final parameter estimates for the POPPK model are presented in Table B.

**Table B. Parameter Estimates for the Final Model**

| **Parameter** | **Estimate** | **SD** | **95% CI** |
| --- | --- | --- | --- |
| ka | 3.48 | 0.200 | (3.09, 3.87) |
| CL/F | 78.1 | 4.02 | (70.3, 86.0) |
| V/F | 119 | 9.80 | (99.7, 138) |
| Q | 0.424 | 0.0435 | (0.339, 0.509) |
| Vp/F | 3.54 | 0.217 | (3.12, 3.97) |
| Dose on CL/F | 0.666 | 0.0127 | (0.641, 0.691) |
| Food on ka | –0.613 | 0.0185 | (–0.649, –0.577) |
| RA on CL/F | 0.625 | 0.0446 | (0.564, 0.739) |
| RUV-HP | 0.166 | 0.00472 | (0.157, 0.175) |
| RUV-RA | 0.860 | 0.0427 | (0.776, 0.994) |
| IIV-CL/F | 0.156 | 0.0409 | (0.0763, 0.237) |
| IIV-V/F | 0.280 | 0.05553 | (0.171, 0.388) |
| Cov CL/F-V/F | 0.173 | 0.0485 | (0.07794 – 0.268) |

CL/F apparent clearance; HP, healthy participants; ka, first-order absorption rate constant; IIV, inter-individual variance; Q, inter-compartmental clearance; RA, rheumatoid arthritis; RUV-HP, random unexplained variability for healthy participants; RUV-RA, random unexplained variability for patients with RA; V/F, apparent volume of distribution; Vp/F, apparent peripheral volume.

**Final Model Control Stream**

**$PROBLEM** run12.mod

**$INPUT** C PROT ID AMT DOSE DFREQ FORM FOOD PERD NTPD TIME FLAG AGE SEX RACE BWT CONC CMT MDV EVID DV

**$DATA** B798ComboNonmemCout.csv

IGNORE=@

**$SUBROUTINE** ADVAN4 TRANS4

**$PK**

STRT = DOSE + DFREQ/10

TRT = DOSE*DFREQ ; total dose

FLG1 = 1 ; nonlinear CL with dose

**IF** (TRT.GT.100) FLG1 = 0

FLG2 = 1; RA study 1006 on CL

**IF** (PROT.EQ.1006) FLG2 = 0

FLG3 = 1 ; known FED status Fast or some fed

**IF** (FOOD.NE.0) FLG3 = 0

TVKA=**THETA**(1)*(1 + (1-FLG3)***THETA**(9));

KA=TVKA

TVCL=(**THETA**(2)*FLG1 + **THETA**(2)***THETA**(7)*(1-FLG1))*(BWT/75)**0.75

TVCL = TVCL * (FLG2 + **THETA**(10)*(1-FLG2))

CL=TVCL***EXP**(**ETA**(1))

TVV2 = **THETA**(3)*(BWT/75)

V2=TVV2***EXP**(**ETA**(2))

Q=**THETA**(4)

V3=**THETA**(5)

S2=V2/1000

ALAG1=**THETA**(6) + (1-FLG3)***THETA**(8)

**$ERROR**

**IF** (F.GT.0) **THEN**

Y=**LOG**(F+0.000001)+(FLG2***EPS**(1) + (1-FLG2)***EPS**(2))

IPRED=**LOG**(F+0.00001)

IRES=DV-IPRED

IWRES=IRES/IPRED

**ELSE**

Y=0

IPRED=0

IRES=0

IWRES=0

**ENDIF**

**$THETA**

(0, 7.5);1 KA

(0, 80) ;2 CL

(0, 125) ;3 V2

(0, 0.4);4Q

(0, 4.5) ;5 ; V3

0 FIX; (0, 0.2) ;6 ALAG

(0, 0.7) ;7 Total Dose CL

0 FIX; (0.01) ;8 Food on ALAG

(-1, 0.1) ;9 Food on KA

(0, 0.8) ;10 RA 1006 on CL

**$OMEGA** **BLOCK**(2)

0.2 ; VAR(CL)

0.1 0.15 ;VAR(V)

**$SIGMA**

0.15 ;sigma Phase 1

0.7 ;sigma RA

**$EST** MAXEVAL=9999 PRINT=5 METHOD=1 INTERACTION NSIG=3 SIGL=6 FILE=run12.ext ;NOTHETABOUNDTEST NOOMEGABOUNDTEST NOSIGMABOUNDTEST

**$COV** PRINT=E MATRIX=S UNCONDITIONAL SIGL=9

**$TABLE** ID DOSE PERD TIME AMT EVID DFREQ MDV CONC DV IPRED CWRES CL V2 KA Q V3 FORM FOOD SEX BWT PROT ETA1 ETA2 NOPRINT ONEHEADER FILE=JAK3_output1.csv FORMAT=,1PE17.9E3

**$TABLE** ID TIME DOSE FOOD STRT PERD FORM DFREQ EVID CONC DV PRED IPRED CWRES ETA1 ETA2 BWT CL V2 KA Q V3 DV NOPRINT ONEHEADER FILE=run12.tab

**$TABLE** PROT ID DOSE CL V2 KA Q V3 NOPRINT ONEHEADER FIRSTONLY FILE=B798_OUTPUT12.txt

**Supplementary Tables and Figures**

**Table S1.** Study 1: Incidence of Treatment-emergent Adverse Events by Preferred Term – All-Causality and Treatment-Related^a^

|  | **Moderate hepatic impairment**  **(n=10)** | | | **Normal hepatic function**  **(n=8)** | | | **Total**  **(n=18)** | | |
| --- | --- | --- | --- | --- | --- | --- | --- | --- | --- |
| **Number of participants by preferred term, n (%)** | **All-causality** | **Treatment-related** | **All-causality** | | **Treatment-related** | **All-causality** | | **Treatment-related** |  |
| With any adverse event | 4 (40.0) | 4 (40.0) | 2 (25.0) | | 1 (12.5) | 6 (33.3) | | 5 (27.8) |  |
| Thrombocytopenia | 1 (10.0) | 1 (10.0)^b^ | 0 | | 0 | 1 (5.6) | | 1 (5.6) |  |
| Diarrhea | 1 (10.0) | 1 (10.0) | 0 | | 0 | 1 (5.6) | | 1 (5.6) |  |
| Cholestasis | 1 (10.0) | 1 (10.0) | 0 | | 0 | 1 (5.6) | | 1 (5.6) |  |
| Arthropod bite | 0 | 0 | 1 (12.5) | | 0 | 1 (5.6) | | 0 |  |
| Hepatic enzyme increased | 1 (10.0) | 1 (10.0)^c^ | 0 | | 0 | 1 (5.6) | | 1 (5.6) |  |
| Headache | 1 (10.0) | 1 (10.0) | 1 (12.5) | | 1 (12.5) | 2 (11.1) | | 2 (11.1) |  |

MedDRA v22.1 coding dictionary was applied. Participants were only counted once per hepatic function group per event. If a participant had more than one occurrence in the same Preferred Term event category, only the most severe occurrence was counted.

^a^An adverse event was considered as a treatment-emergent adverse event if it started after study treatment and before last visit.

^b^One participant experienced worsening of thrombocytopenia after ritlecitinib 30 mg QD on Day 5 (platelet count: 95 × 10^9^/L, relative to 124 × 10^9^/L at baseline), which lead to discontinuation of study drug.

^c^One participant experienced elevated hepatic enzymes (ALT and AST) after ritlecitinib 30 mg QD on Day 5 (ALT and AST: 204 U/L and 126 U/L, relative to 69 U/L and 51 U/L at baseline, respectively), which lead to discontinuation of study drug.

ALT, alanine aminotransferase; AST, aspartate aminotransferase; MedDRA, Medical Dictionary for Regulatory Activities; QD, once daily

**Table S2.** Study 2: Incidence of Treatment-emergent Adverse Events by Preferred Term – All-Causality and Treatment-Related^a^

|  | **Severe renal impairment (n=8)** | |
| --- | --- | --- |
| **Number of participants by preferred term, (n)** | **All-causality** | **Treatment-related** |
| With any adverse event | 3 | 2 |
| Dry eye | 1 | 0 |
| ALT increased | 1 | 1 |
| AST increased | 1 | 1 |
| Pain in extremity | 1 | 0 |
| Dizziness | 1 | 1 |
| Epistaxis | 1 | 0 |

MedDRA v23.1 coding dictionary was applied. Includes all data collected since the first dose of study drug. Participants were counted only once per treatment per event.

^a^An adverse event was considered as a treatment-emergent adverse event if it started after study treatment and before last visit.

ALT, alanine aminotransferase; AST, aspartate aminotransferase; MedDRA, Medical Dictionary for Regulatory Activities

**Figure S1**. Diagnostic plots of population predicted, individual predicted, and CWRES for fit-for-purpose population PK model.


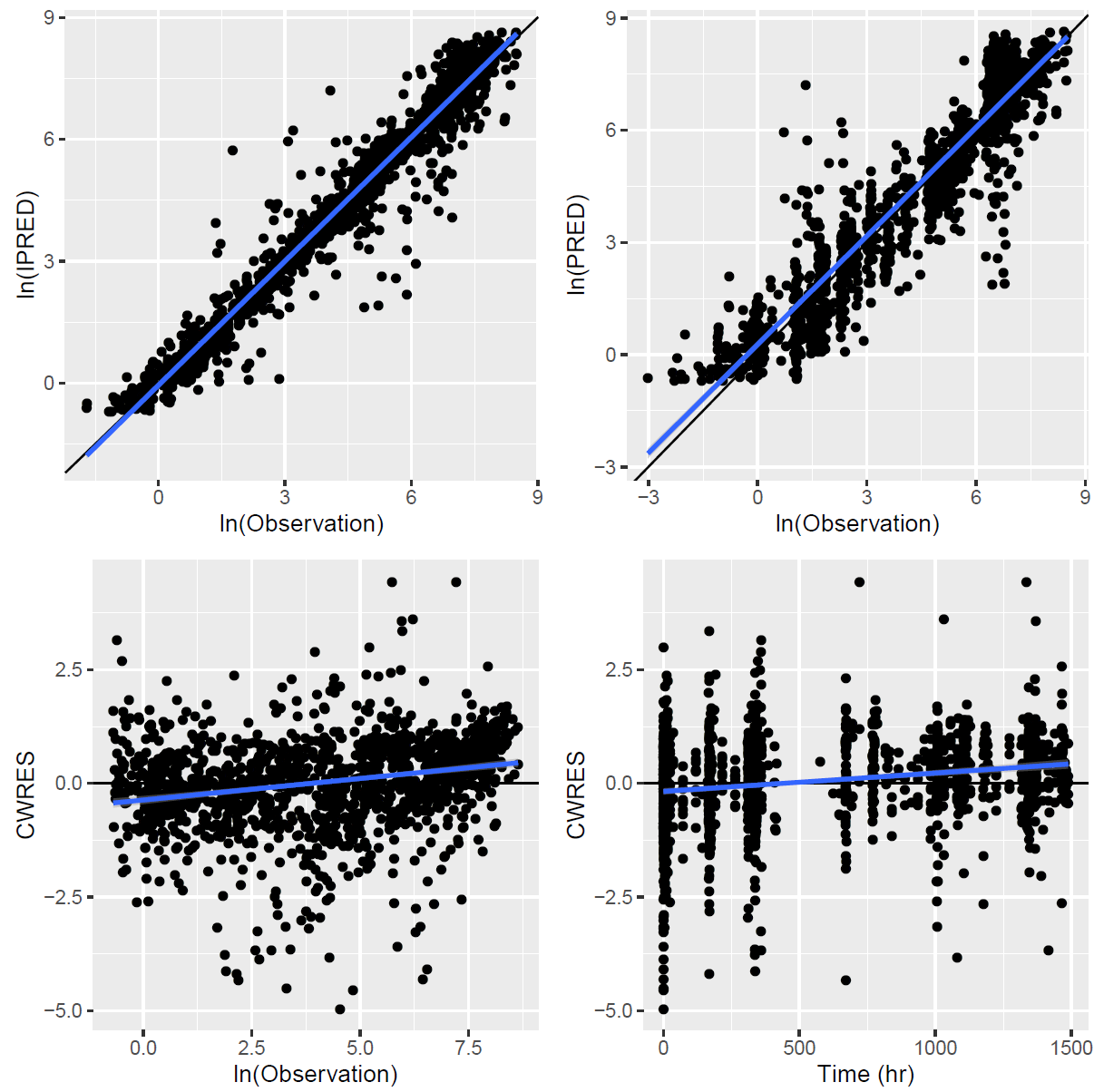


**Figure S2**. Density and Q-Q plots for random effect parameters ETA1 (CL/F) and ETA2 (Vc/F).


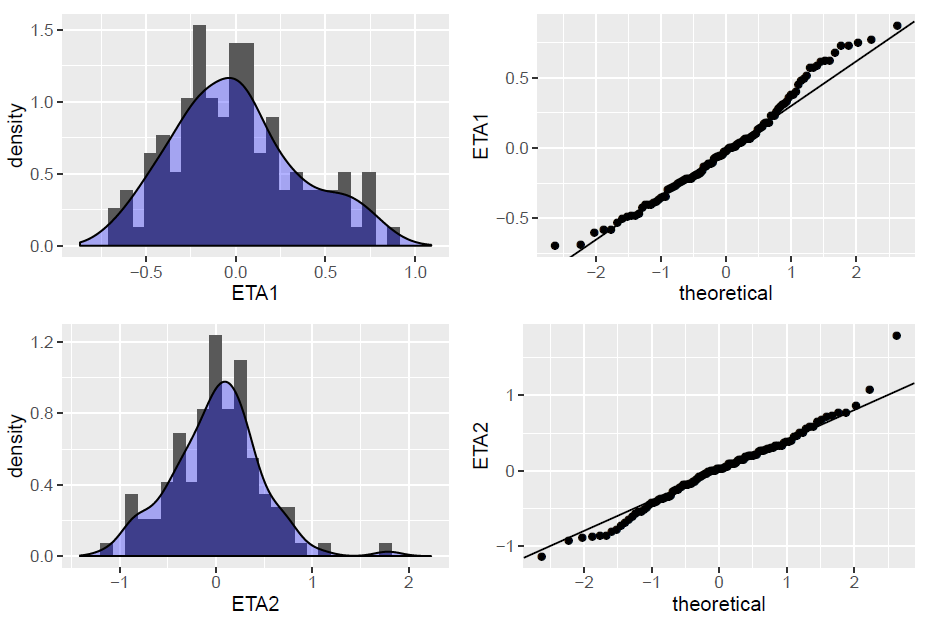


**Figure S3.** Visual predictive checks stratified by dose and time.

1. 5 mg single dose


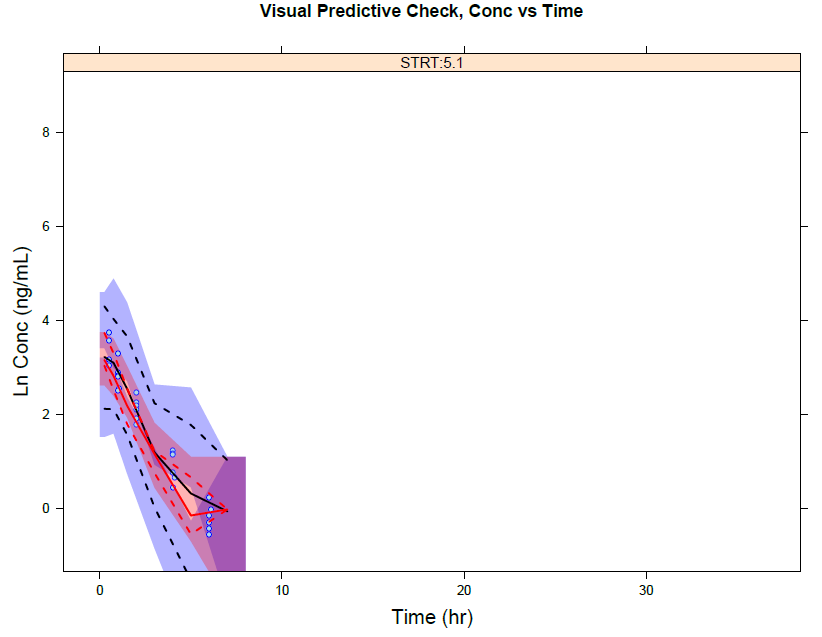


1. 20 mg single dose
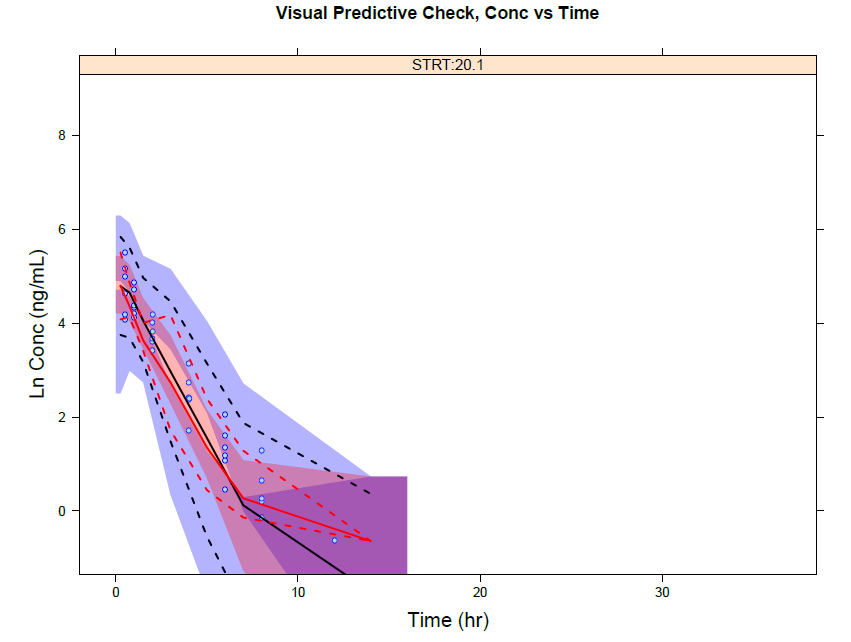

2. 50 mg single dose
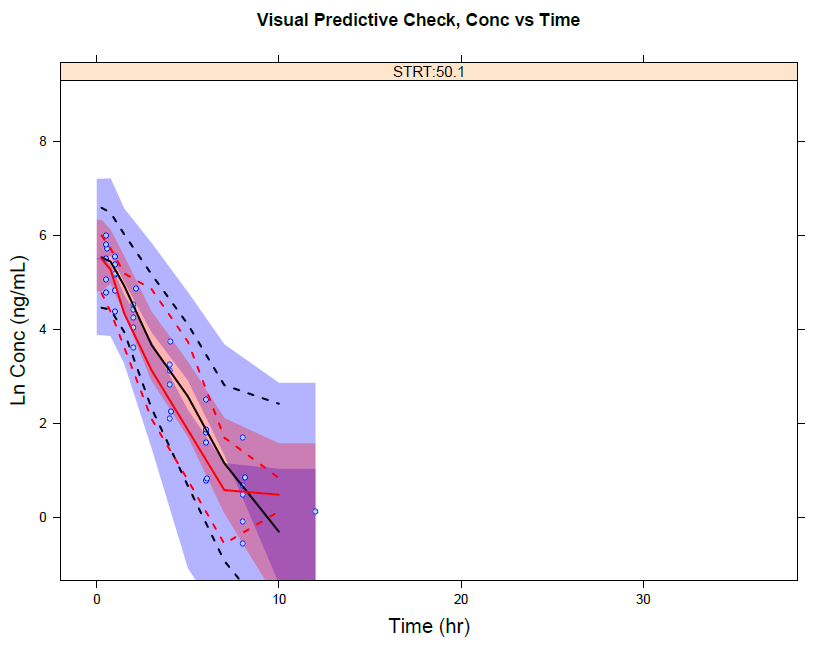

3. 100 mg single dose
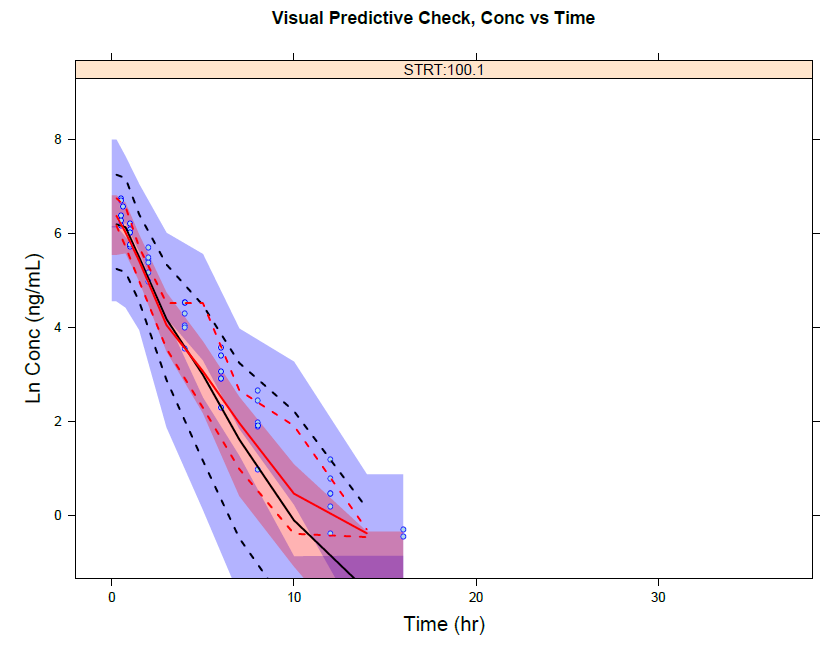

4. 200 mg single dose
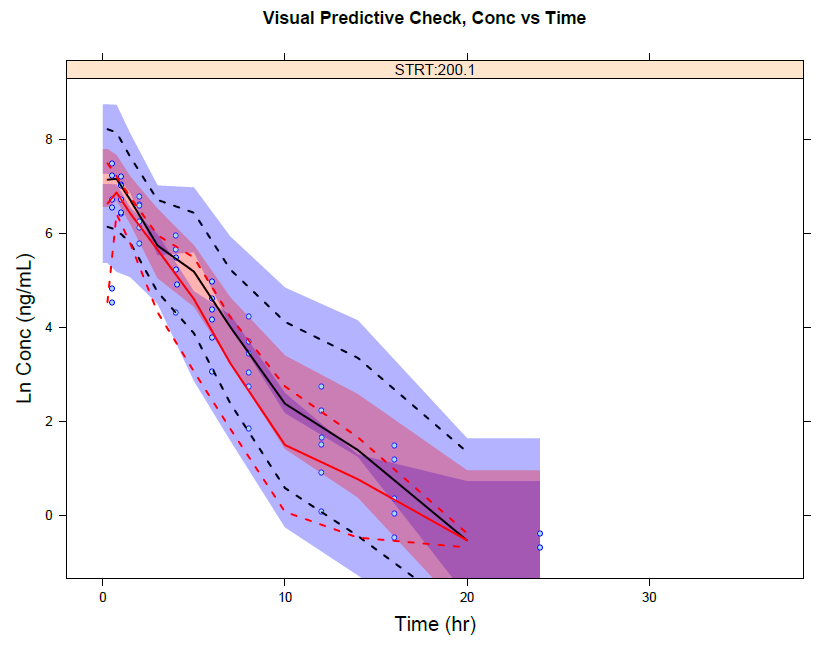

5. 400 mg single dose
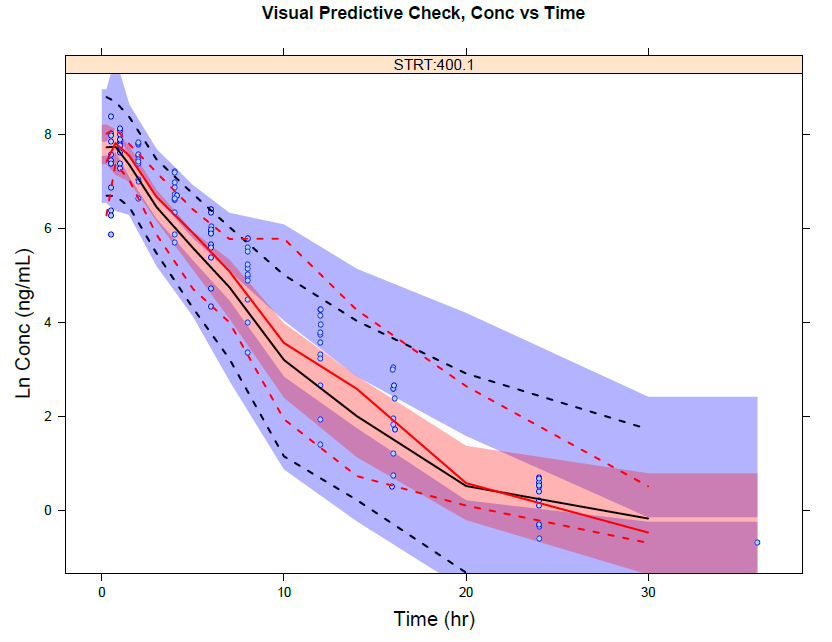

6. 50 mg at steady state
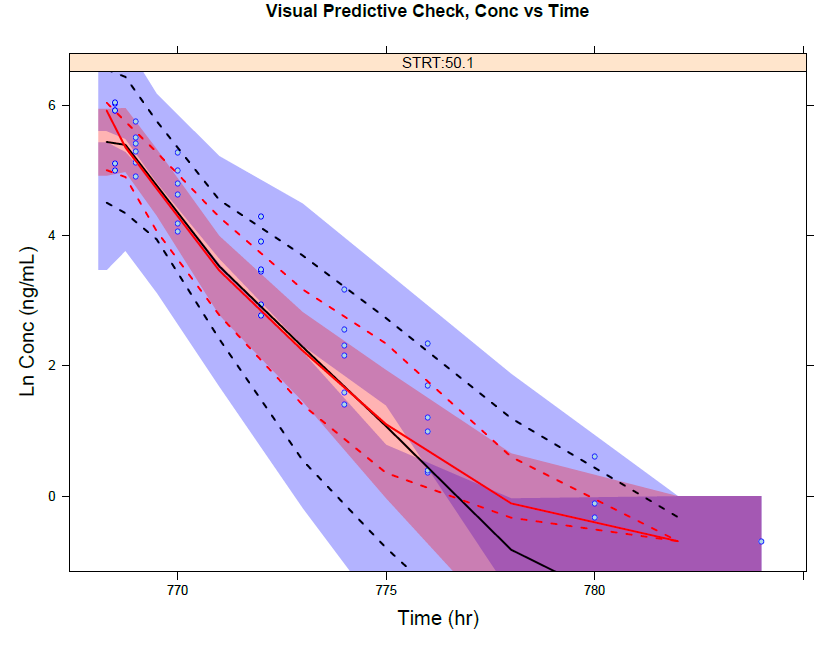

7. 200 mg at steady-state
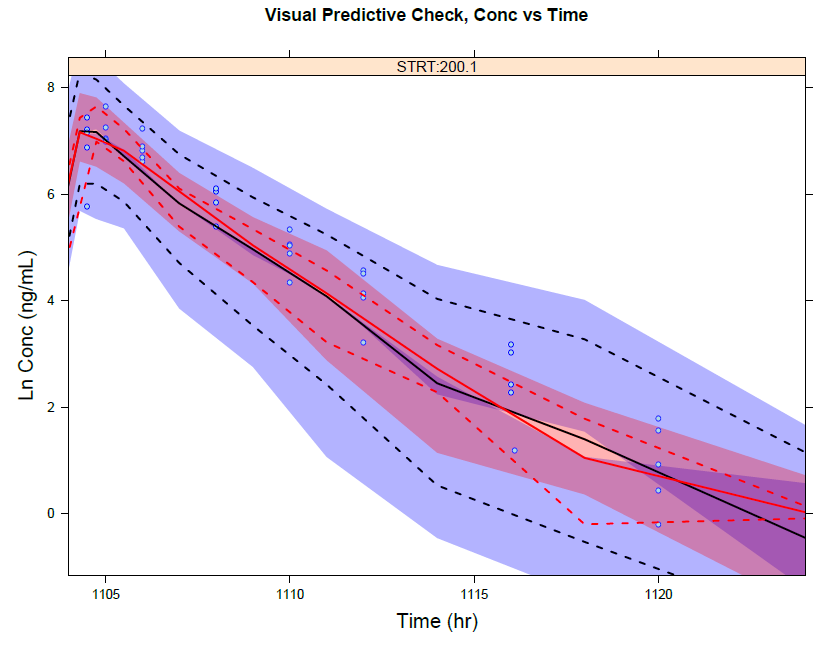


**Figure S4.** Distribution of Geometric Mean Ratios of Moderate Hepatic Impairment Versus Healthy Participants (Study 1)

Histograms represent the distributions of the geometric mean ratios (middle panel), lower 90% CI (top panel), and upper 90% CI (bottom panel) of moderate hepatic impairment versus healthy participants based on ANOVA for 1000 trials. Vertical dashed lines represent the mean of the distribution.

ANOVA, analysis of variance; AUC0-24, area under the plasma concentration–time curve for 24-hour dosing interval; CI, confidence interval; Cmax, maximum plasma concentration.

**Figure S5.** Distribution of Geometric Mean Ratios of Severe Renal Impairment (Study 2) Versus Healthy Participants (Study 1)

Histograms represent the distributions of the geometric mean ratios (middle panel), lower 90% CI (top panel), and upper 90% CI (bottom panel) of severe renal impairment versus healthy participants based on ANOVA for 1000 trials. Vertical dashed lines represent the mean of the distribution.

ANOVA, analysis of variance; AUC0-24, area under the plasma concentration–time curve for 24-hour dosing interval; CI, confidence interval; Cmax, maximum plasma concentration.
